# Supplementary material for: Identity management in the face of HIV and intersecting stigmas: A metasynthesis of qualitative reports from sub-Saharan Africa
Source: PLOS Glob Public Health. 2023 Feb 1;3(2):e0000706. doi: 10.1371/journal.pgph.0000706 (PMC10022386; doi:10.1371/journal.pgph.0000706)
Supplement: S1 Table — (DOCX) [file pgph.0000706.s001.docx]

**S1 Table. Sample Search Strategy - PubMed**

| Sample search strategy – Pub Med |
| --- |
| (("Social Stigma"[Mesh] OR stigma* [tiab]) AND ("Africa South of the Sahara"[Mesh] OR "Central Africa" [tiab] OR Cameroon [tiab] OR "Central African Republic" [tiab] OR Chad [tiab] OR Congo [tiab] OR "Democratic Republic of the Congo" [tiab] OR "Equatorial Guinea" [tiab] OR Gabon [tiab] OR "Eastern Africa" [tiab] OR Burundi [tiab] OR Djibouti [tiab] OR Eritrea [tiab] OR Ethiopia [tiab] OR Kenya [tiab] OR Rwanda [tiab] OR "South Sudan" [tiab] [tiab] OR Tanzania [tiab] OR Uganda [tiab] OR "Southern Africa" [tiab] OR Angola [tiab] OR Botswana [tiab] OR Lesotho [tiab] OR Malawi [tiab] OR Mozambique [tiab] OR Namibia [tiab] OR "South Africa" [tiab] OR Swaziland [tiab] OR eSwatini [tiab] OR Zambia [tiab] OR Zimbabwe [tiab] OR "Western Africa" [tiab] OR Benin [tiab] OR "Burkina Faso" [tiab] OR "Cape Verde" [tiab] OR "Cote d'Ivoire" [tiab] OR Gambia [tiab] OR Ghana [tiab] OR Guinea [tiab] OR "Guinea-Bissau" [tiab] OR Liberia [tiab] OR Mali [tiab] OR Mauritania [tiab] OR Niger [tiab] OR Nigeria [tiab] OR Senegal [tiab] OR "Sierra Leone" [tiab] OR Togo [tiab])) AND (dual [tiab] OR intersectional* OR "inter-sectional*" OR double* [tiab] OR layer* [tiab] OR multilayer* OR multi-layer*) |
| (("Social Stigma"[Mesh] OR stigma* [tiab]) AND ("Africa South of the Sahara"[Mesh] OR "Central Africa" [tiab] OR Cameroon [tiab] OR "Central African Republic" [tiab] OR Chad [tiab] OR Congo [tiab] OR "Democratic Republic of the Congo" [tiab] OR "Equatorial Guinea" [tiab] OR Gabon [tiab] OR "Eastern Africa" [tiab] OR Burundi [tiab] OR Djibouti [tiab] OR Eritrea [tiab] OR Ethiopia [tiab] OR Kenya [tiab] OR Rwanda [tiab] OR "South Sudan" [tiab] [tiab] OR Tanzania [tiab] OR Uganda [tiab] OR "Southern Africa" [tiab] OR Angola [tiab] OR Botswana [tiab] OR Lesotho [tiab] OR Malawi [tiab] OR Mozambique [tiab] OR Namibia [tiab] OR "South Africa" [tiab] OR Swaziland [tiab] OR eSwatini [tiab] OR Zambia [tiab] OR Zimbabwe [tiab] OR "Western Africa" [tiab] OR Benin [tiab] OR "Burkina Faso" [tiab] OR "Cape Verde" [tiab] OR "Cote d'Ivoire" [tiab] OR Gambia [tiab] OR Ghana [tiab] OR Guinea [tiab] OR "Guinea-Bissau" [tiab] OR Liberia [tiab] OR Mali [tiab] OR Mauritania [tiab] OR Niger [tiab] OR Nigeria [tiab] OR Senegal [tiab] OR "Sierra Leone" [tiab] OR Togo [tiab])) AND ("Tuberculosis"[Mesh] OR tuberculos* [tw] OR tuberculin* [tw] OR hiv infections[mh] OR hiv[mh] OR hiv[tw] OR hiv-1[tw] OR hiv-2[tw] OR hiv1[tw] OR hiv2[tw] OR hiv infect*[tw] OR human immunodeficiency virus[tw] OR human immunodeficiency virus[tw] OR human immune-deficiency virus[tw] OR human immune-deficiency virus[tw] OR ((human immun*) AND (deficiency virus[tw])) OR acquired immunodeficiency syndrome[tw] OR acquired immunodeficiency syndrome[tw] OR acquired immuno-deficiency syndrome[tw] OR acquired immune-deficiency syndrome[tw] OR ((acquired immun*) AND (deficiency syndrome[tw]))) |
| (("Social Stigma"[Mesh] OR stigma* [tiab]) AND (South Africa)) AND ("Tuberculosis"[Mesh] OR tuberculos* [tw] OR tuberculin* [tw] OR ((hepatitis c OR hep c OR HCV OR hepatitis C virus) OR (hepatitis b OR hep b OR HBV OR hepatitis B virus)) OR (hpv OR human papilloma virus OR human papillomavirus) OR hiv infections[mh] OR hiv[mh] OR hiv[tw] OR hiv-1[tw] OR hiv-2[tw] OR hiv1[tw] OR hiv2[tw] OR hiv infect*[tw] OR human immunodeficiency virus[tw] OR human immunodeficiency virus[tw] OR human immune-deficiency virus[tw] OR human immune-deficiency virus[tw] OR ((human immun*) AND (deficiency virus[tw])) OR acquired immunodeficiency syndrome[tw] OR acquired immunodeficiency syndrome[tw] OR acquired immuno-deficiency syndrome[tw] OR acquired immune-deficiency syndrome[tw] OR ((acquired immun*) AND (deficiency syndrome[tw]))) |
| (("Social Stigma"[Mesh] OR stigma* [tiab]) AND (South Africa)) AND (dual [tiab] OR intersectional* OR "inter-sectional*" OR double* [tiab] OR layer* [tiab] OR multilayer* OR multi-layer*) |
