# Supplementary material for: Identity management in the face of HIV and intersecting stigmas: A metasynthesis of qualitative reports from sub-Saharan Africa
Source: PLOS Glob Public Health. 2023 Feb 1;3(2):e0000706. doi: 10.1371/journal.pgph.0000706 (PMC10022386; doi:10.1371/journal.pgph.0000706)
Supplement: S1 Text — (DOCX) [file pgph.0000706.s002.docx]

**S1 Text. Articles included in this metasynthesis.**

38. Angwenyi V, Aantjes C, Kajumi M, De Man J, Criel B, Bunders-Aelen J. Patients experiences of self-management and strategies for dealing with chronic conditions in rural Malawi. PLoS One. 2018;13(7):e0199977.

39. Becker TD, Ho-Foster AR, Poku OB, Marobela S, Mehta H, Cao DTX, et al. “It’s When the Trees Blossom”: Explanatory Beliefs, Stigma, and Mental Illness in the Context of HIV in Botswana. Qual Health Res. 2019;29(11):1566–80.

40. Brown A, Wood L. A critical participatory pedagogical approach to enabling life orientation students to develop social literacy through HIV education. Afr J AIDS Res. 2018;17(2):153–62.

41. Buregyeya E, Nuwaha F, Wanyenze RK, Mitchell EMH, Criel B, Verver S, et al. Utilization of HIV and Tuberculosis Services by Health Care Workers in Uganda: Implications for Occupational Health Policies and Implementation. PLoS One. 2012;7(10):e46069.

42. Chileshe M, Bond VA. Barriers and outcomes: TB patients co-infected with HIV accessing antiretroviral therapy in rural Zambia. AIDS Care - Psychol Socio-Medical Asp AIDS/HIV. 2010;22(SUPPL. 1):51–9.

43. Crankshaw T, Voce A, King R, Giddy J, Sheon N, Butler L. Double Disclosure Bind: Complexities of Communicating an HIV Diagnosis in the Context of Unintended Pregnancy in Durban, South Africa. AIDS Behav. 2014;18:53–9.

44. Daftary A, Padayatchi N, Padilla M. HIV testing and disclosure: a qualitative analysis of TB patients in South Africa. AIDS Care. 2007/04/25. 2007;19(4):572–7.

45. Daftary A. HIV and tuberculosis: The construction and management of double stigma. Soc Sci Med. 2012;74(10):1512–9.

46. Daftary A, Padayatchi N. Social constraints to TB/HIV healthcare: Accounts from coinfected patients in South Africa. AIDS Care. 2012;24(12):1480–6.

47. Ezeanolue EE, Iheanacho T, Adedeji IA, Itanyi IU, Olakunde B, Patel D, et al. Opportunities and challenges to integrating mental health into HIV programs in a low- and middle-income country: insights from the Nigeria implementation science Alliance. BMC Health Serv Res. 2020;20.

48. Finnie RKC, Mabunda T, Khoza LB, van den Borne B, Selwyn B, Mullen PD. Pilot study to develop a rapid assessment of tuberculosis care-seeking and adherence practices in rural Limpopo Province, South Africa. Int Q Community Health Educ. 2010;31(1):3–19.

49. Freeman E. Neither “foolish” nor “finished”: identity control among older adults with HIV in rural Malawi. Sociol Health Illn. 2017;39(5):711–25.

50. Gebremariam MK, Bjune GA, Frich JC. Barriers and facilitators of adherence to TB treatment in patients on concomitant TB and HIV treatment: a qualitative study. BMC Public Health. 2010;10:651.

51. Gnauck K, Ruiz J, Kellett N, Sussman A, Sullivan MA, Montoya M, et al. Economic empowerment and AIDS-related stigma in rural Kenya: a double-edged sword? Cult Health Sex. 2013;15(7):851–65.

52. Jani N, Mathur S, Kahabuka C, Makyao N, Pilgrim N. Relationship dynamics and anticipated stigma: Key considerations for PrEP use among Tanzanian adolescent girls and young women and male partners. PLoS One. 2021;16(2):e0246717.

53. Kellett NC, Gnauck K. The intersection of antiretroviral therapy, peer support programmes, and economic empowerment with HIV stigma among HIV-positive women in West Nile Uganda. African J AIDS Res. 2016;15(4):341–8.

54. Kennedy CE, Baral SD, Fielding-Miller R, Adams D, Dludlu P, Sithole B, et al. “They are human beings, they are Swazi”: intersecting stigmas and the positive health, dignity and prevention needs of HIV-positive men who have sex with men in Swaziland. J Int AIDS Soc. 2013;16 Suppl 3:18749.

55. King R, Nanteza J, Sebyala Z, Bbaale J, Sande E, Poteat T, et al. HIV and transgender women in Kampala, Uganda - Double Jeopardy. Cult Health Sex. 2019;21(6):727–40.

56. Kuteesa MO, Seeley J, Cumming RG, Negin J. Older people living with HIV in Uganda: Understanding their experience and needs. African J AIDS Res. 2012;11(4):295–305.

57. Kyakuwa M. Ethnographic experiences of HIV-positive nurses in managing stigma at a clinic in rural Uganda. African J AIDS Res. 2009;8(3):367–78.

58. Kyakuwa M, Hardon A, Goldstein Z. “The Adopted Children of ART”: expert clients and role tensions in ART provision in Uganda. Med Anthropol. 2012;31(2):149–61.

59. LeMasters K, Dussault J, Barrington C, Bengtson A, Gaynes B, Go V, et al. “Pain in my heart”: Understanding perinatal depression among women living with HIV in Malawi. PLoS One. 2020;15(6):e0227935.

60. Logie CH, Okumu M, Musoke DK, Hakiza R, Mwima S, Kyambadde P, et al. Intersecting stigma and HIV testing practices among urban refugee adolescents and youth in Kampala, Uganda: qualitative findings. 2021;

61. Magidson JF, Joska JA, Regenauer KS, Satinsky E, Andersen LS, Seitz-Brown CJ, et al. “Someone who is in this thing that I am suffering from”: The role of peers and other facilitators for task sharing substance use treatment in South African HIV care. Int J Drug Policy. 2019;70:61–9.

62. Matima R, Murphy K, Levitt NS, BeLue R, Oni T. A qualitative study on the experiences and perspectives of public sector patients in Cape Town in managing the workload of demands of HIV and type 2 diabetes multimorbidity. PLoS One. 2018;13(3):e0194191.

63. Matlho K, Lebelonyane R, Driscoll T, Negin J. Policy-maker attitudes to the ageing of the HIV cohort in Botswana. SAHARA J J Soc Asp HIV/AIDS Res Alliance. 2017;14(1):31–7.

64. Mburu G, Ram M, Siu G, Bitira D, Skovdal M, Holland P. Intersectionality of HIV stigma and masculinity in eastern Uganda: implications for involving men in HIV programmes. BMC Public Health. 2014;14(1):1061.

65. Mugisha J, Kinyanda E, Osafo J, Nalukenge W, Knizek BL. Health care professionals’ perspectives on barriers to treatment seeking for formal health services among orphan children and adolescents with HIV/AIDS and mental distress in a rural district in central, Uganda. Child Adolesc Psychiatry Ment Health. 2020;14:26.

66. Njozing N, Edin K, Hurtig A-K. ‘When I get better I will do the test’: Facilitators and barriers to HIV testing in Northwest Region of Cameroon with implications for TB and HIV/AIDS control programmes | SAHARA-J: Journal of Social Aspects of HIV/AIDS. SAHARAH-J J Soc Asp HIV/AIDS. 2010;7(4).

67. Owusu AY. A gendered analysis of living with HIV/AIDS in the Eastern Region of Ghana. BMC Public Health. 2020;20(1):751.

68. Russell SG, Lim S, Kim P, Morse S. The legacy of gender-based violence and HIV/AIDS in the postgenocide era: Stories from women in Rwanda. Health Care Women Int. 2016;37(7):721–43.

69. Regenauer KS, Myers B, Batchelder AW, Magidson JF. “That person stopped being human”: Intersecting HIV and substance use stigma among patients and providers in South Africa. Drug Alcohol Depend. 2020;216.

70. Tokwe L, Naidoo JR. Lived experiences of human immunodeficiency virus and hypertension in the Eastern Cape, South Africa. African J Prim Heal care Fam Med. 2020;12(1):e1–8.

71. Tsang EY ha, Qiao S, Wilkinson JS, Fung AL chu, Lipeleke F, Li X. Multilayered Stigma and Vulnerabilities for HIV Infection and Transmission: A Qualitative Study on Male Sex Workers in Zimbabwe. Am J Mens Health. 2019;13.
