# Supplementary material for: Identity management in the face of HIV and intersecting stigmas: A metasynthesis of qualitative reports from sub-Saharan Africa
Source: PLOS Glob Public Health. 2023 Feb 1;3(2):e0000706. doi: 10.1371/journal.pgph.0000706 (PMC10022386; doi:10.1371/journal.pgph.0000706)
Supplement: Attachment — Submitted filename: Reviewer_Response.docx [file pgph.0000706.s003.docx]

Dear Editorial Staff and Esteemed Reviewers,

Please find below a table that includes a response to each point raised by the editors and reviewers. Changes are noted within the manuscript in yellow highlighting and are referenced here with the corresponding line numbers.

| Reviewer comment | Response |
| --- | --- |
| Please amend your detailed Financial Disclosure statement. This is published with the article. It must therefore be completed in full sentences and contain the exact wording you wish to be published. | Thank you – we have included a detailed financial disclosure statement with the required information. |
| Please ensure that the funders and grant numbers match between the Financial Disclosure field and the Funding Information tab in your submission form. Note that the funders must be provided in the same order in both places as well. | This has been updated in the Financial Disclosure field and corresponds with the funding information tab in the submission form. |
| Please update your Competing Interests statement. If you have no competing interests to declare, please state: “The authors have declared that no competing interests exist.” | Thank you, we have included the suggested statement, “The authors have declared that no competing interests exist.” |
| Please amend your Data Availability Statement and indicate where the data may be found. | This is a meta-synthesis of existing publicly available data. All data is available through published peer-reviewed journals and can be found using the citation information. Our data availability statement has been amended to reflect this. |
| Even though the majority of the article focuses on HIV and associated stigma, it is not reflected in the title. | Thank you for this comment. We have updated the title to reflect HIV as well as intersecting stigmas. |
| This paper will benefit if the definitions and concepts behind “Stigma” and “Intersectionality” are laid out first. | We agree that it is essential to have a common working definition for stigma and intersectionality. In lines 32-34 we have added the following: Stigma can be defined as a negatively perceived attribute that precludes an individual from full social acceptance and contributes to social, financial and/or health related inequity.  We would like to bring the reviewer’s attention the second half of the introduction, (lines 38-47) where we offer a nuanced explanation to the complexity of intersectional stigma. |
| Were you open to any infectious disease stigma, or was HIV an inclusion criterion? It is not mentioned as one but it is interesting that all studies addressed it. This also relates to the next comment about search strategy. | For this analysis, we were open to several infectious diseases based on geographic disease burden and the stigma literature, this is now reflected in lines 98-99. Line 97, in the methods section, specifies that one of our inclusion criterion was, “included at least one element of infectious disease stigma”. The methods section now includes examples of specific diseases included in the search strategy.  Because this search was conducted early in the pandemic, COVID19 was not specifically included in the search strategy. Despite the significant stigma associated with Hansen’s disease, we did not include it in the search strategy because we limited our search to sub-Saharan Africa excluding India, Brazil and Pakistan, which represent nearly 90% of global infections.  In response to the comment below we have also included a sample search strategy for in the supplemental tables. |
| It would be useful to either see one or two full examples of the search strategies (perhaps a table or text box), or get a bit more details about how they differed, e.g. by database or what does it mean that a “variety” was used? | Thank you for bringing this up. We have now included the comprehensive search strategies used for PubMed as supplemental material (Table 1).  We also include detail in lines 92-93 about variations on the term “intersectional stigma” intended to maximize recall. |
| The numbers in the PRISMA diagram do not seem to fully add up so some clarification or correction is needed. | Thank you for bringing this to our attention and we apologize for the error. The PRISMA diagram has been updated with the needed corrections. |
| Is “same-sex behaviour” the best term to use here? If you are referring to sexual behaviour, then the word ‘sexual’ is probably missing. It also seems to me that this term is often used in animal studies so I would just make sure that it is considered appropriate for this context. | Thank you, we have reworded the sentence in lines 207-208 for precision and clarity. |
| Your synthesis is very interesting but it is difficult to trace the evidence from the way it is presented with few references. Is there a reason why the supplementary tables cannot be part of the paper itself? I would be in favour of that if journal guidelines allow it as that would help the reader see which studies informed which part of the synthesis. | We agree that the tables should be part of the paper itself. Congruent with Plos Global Public Health journal guidelines the supplemental tables are now part of the main body.  The purpose of qualitative metasynthesis is to go beyond a summary or synthesis of evidence, instead integrating the findings of multiple qualitative sources to extract overarching themes that surpass the meaning of any individual publication in isolation (Sandelowski & Barroso 2007; Lachal, Revah-Levy, Orri & Morro 2017). During the metasynthesis process, we are building an analysis that stands apart from the individual studies. Consistent with the tradition of qualitative metasynthesis, we have chosen not to cite individual studies because the results are reflective not of the original authors’ analyses but our own independent analysis. The results are a wholly new interpretation of the data. If it would help to allay concerns, we are happy to provide exemplars from the work of Barroso and Sandelowski’s published metasyntheses as they also published the *Handbook for Synthesizing Qualitative Research* which outlines the methodology used in this analysis. |
| Line 354 typo: “threated” | Thank you – the typo has been corrected. |
| Line 366 “as” missing from “such as” | Thank you – the typo has been corrected. |
| I am a bit uncomfortable with how the whole of Sub-Saharan Africa is lumped together in explanations about traditional masculinities, can you add some nuance here? Again, being able to trace the evidence would also help as the reader would see where specific statements come from. | While we agree that sub-Saharan Africa is not a monolith, the findings that led to the development of our results and which spurred the discussion were taken from a variety of geographic locations. As a result, we feel that traditional masculinities contribute to a common experience that transcends national borders. We acknowledge that this is not the experience of all men, but of many. However, we have attempted to bring some additional nuance and clarification to the discussion section so that we do not appear to endorse a singular experience (Lines 362, 364-367). |

Respectfully,

Authors - PGPH-D-22-00449

Identity Management in the Face of HIV and Intersecting Stigmas: A Metasynthesis of Qualitative Reports from sub-Saharan Africa
